# Reduced Level of Tear Antimicrobial and Immunomodulatory Proteins as a Possible Reason for Higher Ocular Infections in Diabetic Patients

**DOI:** 10.3390/pathogens10070883

**Published:** 2021-07-12

**Authors:** Gergő Kalló, Anita Katalin Varga, Judit Szabó, Miklós Emri, József Tőzsér, Adrienne Csutak, Éva Csősz

**Affiliations:** 1 Proteomics Core Facility, Department of Biochemistry and Molecular Biology, Faculty of Medicine, University of Debrecen, Egyetem tér 1, 4032 Debrecen, Hungary; kallo.gergo@med.unideb.hu (G.K.); vargaanitakatalin@gmail.com (A.K.V.); tozser@med.unideb.hu (J.T.); 2 Biomarker Research Group, Department of Biochemistry and Molecular Biology, Faculty of Medicine, University of Debrecen, Egyetem tér 1, 4032 Debrecen, Hungary; 3 Department of Medical Microbiology, Faculty of Medicine, University of Debrecen, Nagyerdei krt. 98, 4032 Debrecen, Hungary; szabjud@med.unideb.hu; 4 Department of Medical Imaging, Division of Nuclear Medicine and Translational Imaging Faculty of Medicine, University of Debrecen, Egyetem tér 1, 4032 Debrecen, Hungary; emri.miklos@med.unideb.hu; 5 Laboratory of Retroviral Biochemistry, Department of Biochemistry and Molecular Biology, Faculty of Medicine, University of Debrecen, Egyetem tér 1, 4032 Debrecen, Hungary; 6 Department of Ophthalmology, Faculty of Medicine, University of Debrecen, Egyetem tér 1, 4032 Debrecen, Hungary; csutak.adrienne@pte.hu; 7 Department of Ophthalmology, Faculty of Medicine, University of Pécs, Rákóczi út 2, 7623 Pécs, Hungary

**Keywords:** tear, antimicrobial activity, diabetes mellitus, targeted mass spectrometry, ocular infection, antimicrobial and immunomodulatory protein, AMP

## Abstract

(1) Background: Diabetes mellitus is one of the most common metabolic disorders and a risk factor for bacterial ocular infections. Our aim was to examine the antibacterial activity of tears from patients with diabetes mellitus with and without diabetic retinopathy and to link this activity to the level of tear proteins. (2) Methods: Non-stimulated basal tears were collected from 39 eyes of 35 subjects. The antibacterial activity of tear pools was tested against pathogenic *Staphylococcus aureus* ATCC 29213, *Escherichia coli* ATCC 26922 and *Pseudomonas aeruginosa* ATCC 27853 strains. The levels of 10 antimicrobial and immunomodulatory proteins were analyzed in the individual tear samples of the studied groups by SRM-based targeted mass spectrometry analysis. (3) Results: Disease stage-specific antimicrobial effect was observed in case of *Staphylococcus aureus* ATCC 29213 strain, and a non-disease specific inhibitory effect was observed in case of *Pseudomonas aeruginosa* ATCC 27853 strain. Changes in the levels of the studied antimicrobial and immunomodulatory proteins in the tears of the studied groups were also observed. (4) Conclusions: The higher ocular infection rate observed in diabetic patients may be the consequence of the decreased antimicrobial activity of tears possibly caused by the changes in the levels of antimicrobial and immunomodulatory proteins.

## 1. Introduction

Diabetes mellitus (DM) is one of the most common diseases of the 21st century; the current prevalence is approximately 3% worldwide [1]. DM is a metabolic disorder of multiple etiology characterized by chronic hyperglycemia with alterations of carbohydrate, lipid and protein metabolism due to defects in insulin secretion and/or insulin signal transduction pathway [2]. Patients with diabetes have high risk to develop diabetic retinopathy (DR), which is one of the most common eye-related complication of DM [3]. DR can progress from non-proliferative diabetic retinopathy (NPDR) to more severe proliferative diabetic retinopathy (PDR) [4], finally leading to sight threatening condition or even blindness [5].

Studies have shown that DM is a risk factor for ocular bacterial infections such as infective keratitis [6] and acute infectious conjunctivitis [7]. Pathological changes in the cornea and conjunctiva were noted in most of the patients suffering from DM [8]. These changes included a significant increase in squamous metaplasia, abnormalities in the corneal basement membrane [9] and a reduction in goblet cell density [10,11]. Several morphological changes in the conjunctival blood vessels such as loss of capillaries and macrovessel dilation have also been reported in diabetic patients similar to the well-known vessel changes in the retina [12,13].

Our workgroup has previously described that pathological changes, such as DR [14] and Alzheimer’s disease [15] could change the level of several tear proteins that are part of the chemical barrier system of the eye. These proteins are called antimicrobial and immunomodulatory proteins (AMPs) and are mainly involved in the host defense mechanisms [16]. During the progression of these diseases, the microenvironment of the eye changes; therefore, the protein content of the produced tears will change, altering the chemical barrier of the eye.

Most studies assessing the level of tear proteins have sought to establish a relationship to DR [17,18,19]. Of over 1500 proteins that have been identified in tear fluid [20,21,22] the levels of nerve growth factor, apolipoprotein A1, lipocalin-1, lactotransferrin, lacritin, lysozyme-C, lipophylin A and Ig λ chain have found to be increased in patients with PDR, while lower levels of lipocalin-1, hsp27, β2-microglobulin and increased levels of endothelin and neuron-specific enolase have been found in patients with NPDR [14,23,24,25,26].

Considering the low amount of produced tears, the detection of tear proteins with lower abundance requires highly sensitive and robust techniques such as the different mass spectrometry-based proteomics approaches. In case of “shotgun” techniques, there is no need for prior information about the proteins of interest, but the sensitivity is lower compared to targeted proteomics techniques [27]. In case of targeted proteomics approaches like Selected Reaction Monitoring (SRM), only the proteins of interest will be analyzed providing higher sensitivity [28]. SRM-based mass spectrometry allows qualitative and quantitative analysis of proteins in complex biological samples using stable isotope-labeled (SIL) synthetic peptides [28,29]. The SIL peptides co-elute, ionize and fragment identically with their endogenous counterparts serving both as quality control and as standard for quantification of endogenous peptides [30].

Considering that the tear protein profile changes in DM and DR, and patients with DM have higher risk for ocular infection, we aimed to measure the level of some tear AMPs and to identify a link between the tear protein levels and the antimicrobial activity of tears. The antibacterial activity of tears from the studied groups was tested against common pathogenic bacteria. At the same time, the level of proteins such as Znα2-glycoprotein, prolactin inducible protein, lysozyme-C, lipophylin A, lipocalin-1, lactotransferrin, extracellular glycoprotein lacritin, Ig λ chain C region, galectin 3-binding protein and dermcidin having a role in the host defense was analyzed by SRM-based relative quantification in the tears of healthy controls and patients with DM and DR.

## 2. Results

### 2.1. Antibacterial Activity of Tears

The analysis of the antibacterial activity of tears was investigated by a microtiter plate assay. Tear samples from healthy individuals (Healthy group), patients with Diabetes mellitus without any sign of retinopathy (DM group), patients with DM and non-proliferative diabetic retinopathy (NPDR group) and patients with DM and proliferative diabetic retinopathy (PDR group) were collected. Equal amounts of tear samples from each group were pooled and used for antibacterial activity assay. The effect of the tears was tested against pathogenic *Staphylococcus aureus* ATCC 29213, *Escherichia coli* ATCC 26922 and *Pseudomonas aeruginosa* ATCC 27853 strains; physiological saline treated bacterial cultures were used as controls, and the growth rate of each culture was monitored for 10 h. 

**Figure 1:**
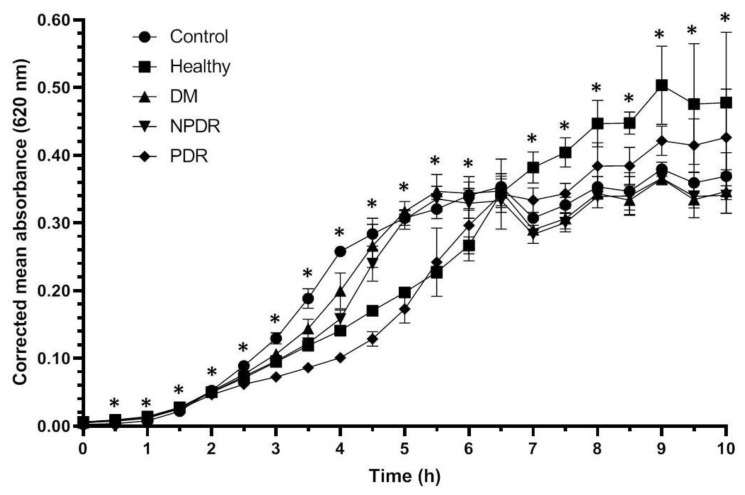
Antibacterial activity of tears against *Staphylococcus aureus* ATCC 29213 strain. The y-axis represents the corrected mean absorbance values (±SD) of three technical replicates while the x-axis shows the time of the experiment in hours. * Indicates significant differences between the groups (*p* < 0.05). The differences between the individual groups are detailed in Table 1.

During the experiment, the *Staphylococcus aureus* ATCC 29213 strain reached the log phase after 1.5 h, and the profile of the growth curve of the bacteria treated with tears was different compared to the control (Figure 1, Table 1, Supplementary Table S1). 

Tear samples were able to inhibit the growth of *Staphylococcus aureus* cultures compared to the physiological saline-treated controls. The tear originating from healthy and diabetic patient groups affected the growth rate of the *Staphylococcus aureus* ATCC 29213 strain. During the log phase, tears from patients with PDR have shown the highest antibacterial activity, while the tears from patients with DM have exerted the lowest antibacterial activity compared to the control strain. During the log phase the growth rate of *Staphylococcus aureus* ATCC 29213 culture was significantly higher in the cultures treated with tears from the DM group compared to the healthy group, indicating the decreased antimicrobial activity of tears of patients with DM. The antimicrobial activity of tears from patients from the NPDR group was significantly lower during the late log phase while the antimicrobial effect of tears from the PDR group was significantly higher compared to the healthy group. Differences were not observed during the comparison of the growth rate of the bacterial cultures treated with tears from the DM and NPDR groups; however, the comparison of the effect of tears from DM and NPDR groups with tears from PDR group showed a significantly elevated antimicrobial activity in the PRD group. After 6.5 h, the bacteria cultures reached the stationary phase, and the inhibitory effect of tears could not be observed; however, significant differences were still observed between the studied groups.

**Figure 2:**
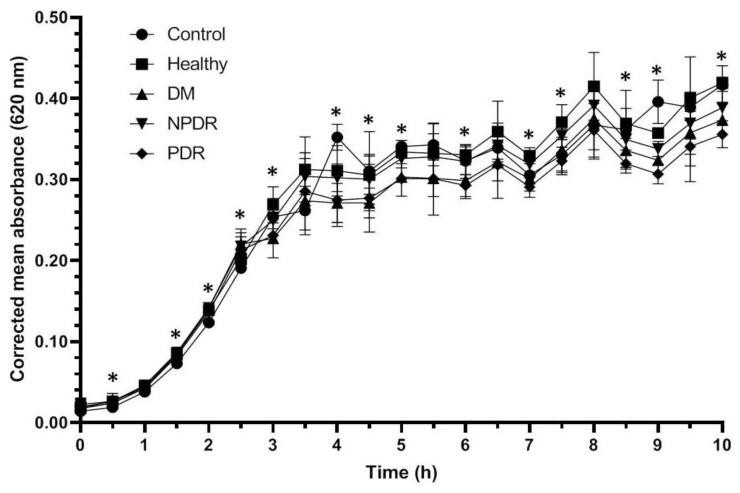
Antibacterial activity of tears against *Escherichia coli* ATCC 26922 strain. The y-axis represent the corrected mean absorbance values (±SD) of three technical replicates while the x-axis shows the time of the experiment in hours. * Indicates significant differences between the groups (*p* < 0.05). The differences between the individual groups are detailed in Table 2.

The growth of the *Escherichia coli* ATCC 26922 cultures reached the log phase after 1 h, and after 3.5 h the cultures reached the stationary phase (Figure 2). The growth rate of the treated bacterial cultures was similar to the controls; however, significant differences were observed between the controls and cultures treated with different tear samples (Table 2, Supplementary Table S2). 

During the log phase the growth rate of the bacterial culture treated with tears was significantly higher compared to the controls, but there was no difference between the growth rate of *Escherichia coli* ATCC 26922 cultures treated with tears. Significant differences were observed between the cultures treated with tears from the studied groups during the stationary phase as well; however, patterns indicating the antimicrobial activity against *Escherichia coli* ATCC 26922 were not observed. Significant differences were observed between the samples treated with tears originating from the different disease groups; however, patterns indicating the effective antimicrobial activity were not observed. 

**Figure 3:**
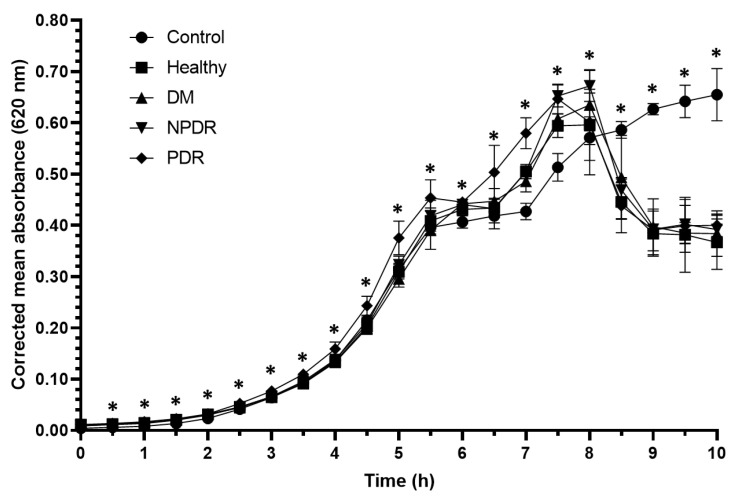
Antibacterial activity of tears against *Pseudomonas aeruginosa* ATCC 27853 strain. The y-axis represent the corrected mean absorbance values (±SD) of three technical replicates while the x-axis shows the time of the experiment in hours. * Indicates significant differences between the groups (*p* < 0.05). The differences between the individual groups are detailed in Table 3.

With the applied experimental setup, the *Pseudomonas aeruginosa* ATCC 27853 strain entered the log phase after 3.5 h (Figure 3), and the applied tears had positive effect on the growth rate of the bacterial cultures (Table 3, Supplementary Table S3). 

In the first 2 h of the experiment, the growth rate of the cultures treated with tears was significantly higher compared to the controls. In case of the cultures treated with tears from the PDR group, the growth rate was significantly higher compared to the controls for 7.5 h. During the log phase, the growth rate of cultures treated with tears from the PDR group was significantly higher compared to the cultures treated with tears from the healthy, DM and NPDR groups. After 8 h the growth rate of the tear-treated cultures was significantly decreased compared to the controls indicating a delayed antimicrobial effect of tears on *Pseudomonas aeruginosa* ATCC 27853 strain.

### 2.2. Changes in the Chemical Barrier Composition of Tears Collected from Patients with Diabetes Mellitus

Our workgroup has previously demonstrated that the level of some AMPs has changed to a statistically significant extent in the tears originating from patients with DM [14] or Alzheimer’s disease [15] compared to controls. The SRM method optimized in our previous study [15] was used in these experiments as well, and the level of Znα2-glycoprotein, prolactin inducible protein, lysozyme-C, lipophylin A, lipocalin-1, lactotransferrin, extracellular glycoprotein lacritin, Ig λ chain C region, galectin 3-binding protein and dermcidin was examined. 

**Figure 4:**
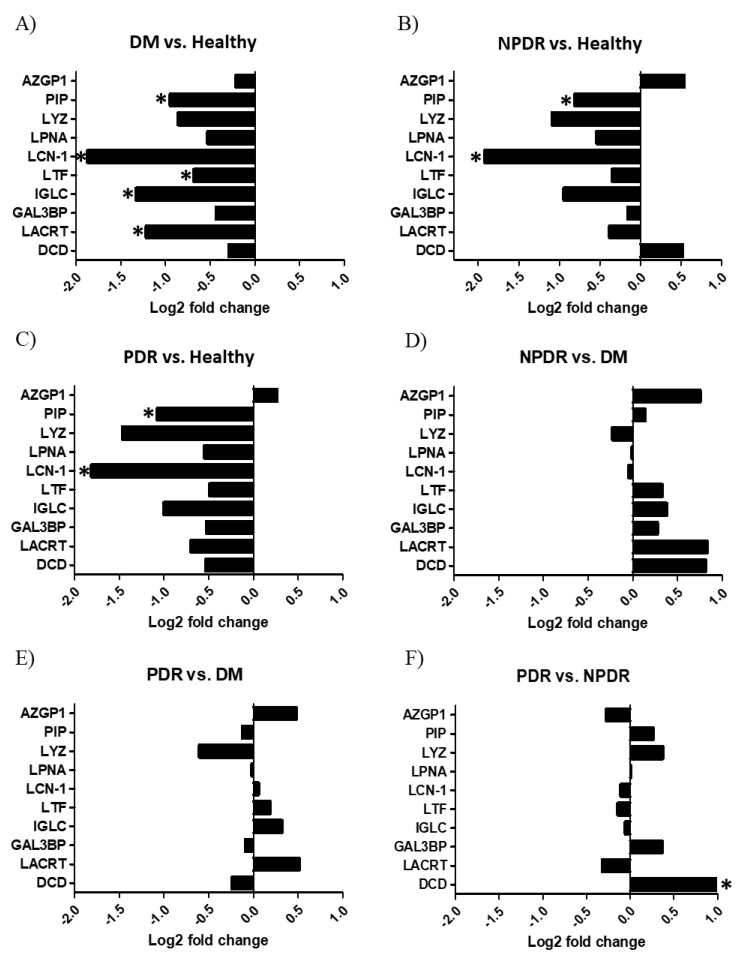
Quantitative analysis of tear proteins by selected reaction monitoring. The log2 fold change of the studied proteins in tears are indicated. (**A**) Tear samples from patients with diabetes without retinopathy (DM) compared to healthy controls. (**B**) Tear samples from patients with non-proliferative diabetic retinopathy (NPDR) compared to the healthy controls. (**C**) Tear samples from patients with proliferative diabetic retinopathy (PDR) compared to the healthy controls. (**D**) Tear samples from NPDR groups compared to the DM group. (**E**) Tear samples from PDR groups compared to the DM group. (**F**) Tear samples from PDR groups compared to the NPDR group. * *p* < 0.05. The proteins are labeled using their gene names; AZGP1: Znα2 glycoprotein, PIP: prolactin-inducible protein, LYZ: lysozyme-C, LPNA: lipophilin A, LCN1: lipocalin-1, LTF: lactotransferrin, IGLC: Ig λ chain C region, LACRT: extracellular glycoprotein lacritin, GAL3BP: galectin 3-binding protein, DCD: dermcidin.

Thirty-nine tear samples were analyzed by SRM-based targeted mass spectrometry. After the evaluation of the registered spectra, the relative quantities of the examined tear proteins were compared in the studied groups (Figure 4). 

The comparison of the healthy control group with the DM group revealed that the level of lipocalin-1, lactotransferrin, extracellular glycoprotein lacritin, prolactin inducible protein and Ig λ-chain C region was significantly lower in patients with DM compared to the healthy group (Figure 4A). The level of lipocalin-1 and prolactin inducible protein was significantly lower in the tear samples of the NPDR group compared to the healthy tears (Figure 4B), and the same tendency was observed in case of the tear samples of the PDR group compared to the healthy individuals (Figure 4C). The comparison of the NPDR and PDR groups with the DM group (Figure 4D,E respectively) showed no significant changes while the comparison of PDR and NPDR groups revealed that the level of dermcidin was significantly higher in the tear samples of the PDR group (Figure 4F).

## 3. Discussion

Tear fluid is a complex mixture of proteins, lipids, salts and other organic molecules produced by the lacrimal glands, Meibomian glands and conjunctival goblet cells. The functions of the tear film are the lubrication of the eye, delivery of nutrients and maintaining of the refractivity of the cornea [31]. Besides these roles, tear creates an effective chemical barrier on the surface of the eye via secreted AMPs which provide protection against pathogens [32]. The higher ocular infection rate of patients with DM [7] may be the consequence of the altered tear protein content. To test this hypothesis, a microtiter plate-based assay was used to examine the antibacterial activity of tears of healthy controls and patients with DM, NPDR and PDR against three pathogenic bacterial strains. 

*Staphylococcus aureus* is a Gram-positive coccus causing the majority of human bacterial infections [33]. It is also the major pathogen of the eye able to infect the tear duct, eyelid, conjunctiva, cornea, anterior and posterior chambers and the vitreous chamber causing ocular infections such as blepharitis, dacryocystitis, conjunctivitis, keratitis and endophthalmitis [34]. *Escherichia coli*, a Gram negative facultative anaerobic bacteria, is the most prevalent commensal inhabitant of the gastrointestinal tracts of humans and warm-blooded animals, as well as one of the most important pathogens [35] causing different ocular infections such as keratitis, conjunctivitis and dacryocystitis [36]. *Pseudomonas aeruginosa* is a Gram-negative ubiquitous environmental bacterium and an opportunistic human pathogen as well [37]. This pathogen is the most frequent isolate of Gram-negative ocular infections capable of causing keratitis, blepharitis, conjunctivitis and blepharoconjunctivitis [36]. Since these three bacteria are common pathogens causing eye infections, the antibacterial activity was tested on pathogenic *Staphylococcus aureus* ATCC 29213, *Escherichia coli* ATCC 26922 and *Pseudomonas aeruginosa* ATCC 27853 strains. The examined bacteria cultures were diluted to approximately the same absorbance as the blank before the experiments and the three bacterial strains have shown different growth curves (Figure 1, Figure 2 and Figure 3). *Staphylococcus aureus, Escherichia coli* and *Pseudomonas* cultures have different generation time as described by M. Mason in the early 1900s [38]. Among the examined bacterial strains *Escherichia coli* had the shortest generation time while *Pseudomonas* had the longest resulting in different growth curves during the experiments. 

Antimicrobial activity against *Staphylococcus aureus* ATCC 29213 strain was observed during the log phase of the growth of the cultures; the tear treatment was effective during a 3 h time period (Figure 1). The applied tear volume was effective for only 6.5 h, and after the antibacterial activity of the tears run out, the bacteria could possibly utilize the tear components as nutrients. Our data suggest that in case of antimicrobial activity against the *Staphylococcus aureus* ATCC 29213 strain, the tears collected from patients with DM were the least effective; however, the tears originating from patients with PDR had the highest antimicrobial activity (Figure 1, Table 1). In case of *Escherichia coli* ATCC 26922 strain, antimicrobial activity was not observed during the experiments (Figure 2, Table 2), while antimicrobial activity was detected at the late phase of the growth of *Pseudomonas aeruginosa* ATCC 27853 strain (Figure 3, Table 3). Significant differences were not observed between the effect of tears originating from patients with DM, NPDR or PDR indicating that the antimicrobial activity of tears on this bacterial strain was diabetes-related and independent of the ocular status. 

We hypothesize that the low effectivity of the tear originating from patients with DM can be a possible consequence of the decreased level of AMPs, and this phenomenon might explain the higher ocular infection rate in patients with DM. Along with the examination of antibacterial activity, the level of some major tear AMPs was analyzed by SRM-based targeted mass spectrometry. Our SRM analyses revealed that the level of prolactin inducible protein, lipocalin-1, lactotransferrin, Ig λ chain C region and extracellular glycoprotein lacritin was significantly decreased in the tears of patients with DM compared to healthy controls (Figure 4A). These proteins are highly abundant tear proteins with various defense functions. It has been shown that lactotransferrin, found in all body fluids, is an active agent against microbes and parasites and has been implicated in protection against cancer [39]. Because of its iron sequestering activity, it has an important role in the prevention of bacterial colonization. Lipocalins are a family of lipid binding proteins with protease inhibitor activity and by sequestrating iron, they can limit bacterial growth [40,41]. Extracellular glycoprotein laritin is a secreted glycoprotein found in tears and saliva. The protein has various functions including effect on lacrimal gland secretion [42], epithelial cell proliferation [43] and corneal wound healing [44]. Additionally, the C-terminal fragment of extracellular glycoprotein lacritin has bactericidal activity [45]. Prolactin-inducible protein is an aspartyl protease enzyme [46], which can be found in various body fluids as part of the host defense system. Beside the protease activity, prolactin-inducible protein can modulate immune reaction by binding to immunoglobulin G and Zn-α-2-glycoprotein [47,48], and its elevated expression has been associated with breast cancer [49]. Due to the decreased level of major tear AMPs pathogenic bacteria may invade the surface of the eye more easily causing ocular infection. At the same time, AMPs were also found to be negative regulators of the generation of reactive oxygen species (ROS) [50] thus, the reduced level of tear AMPs may lead to extensive ROS production resulting in photoreceptor and retinal degradation [51,52].

The most effective antibacterial activity was observed during the log phase in case of the tears from patients with PDR. The SRM analysis revealed that the level of the studied AMPs was higher in the PDR compared to DM, but no statistically significant difference could be observed. The comparison of the level of AMPs between PDR and healthy groups indicated decreased AMP levels in the tears of the PDR group (Figure 4C), while the level of dermcidin was significantly elevated in PDR compared to the NPDR group (Figure 4F). Dermcidin is the main skin AMP, which is also present in tears and exerts broad spectrum antimicrobial activity [39,53]. Dermcidin is constitutively secreted by eccrine sweat glands and epithelial cells, and its secretion cannot be further induced by skin injury or inflammation [54]. The increased expression of dermcidin has been demonstrated in lung, prostate and pancreatic cancer cells [55,56] suggesting its role in proliferative pathological changes. The reason for the highest antimicrobial effect of tears from patients with PDR can be the presence of other proteins, AMPs or other biomolecules which were not analyzed in this study. 

AMPs act as the first line of host defense, and the alteration in the composition of secreted AMPs may drive the invasion of pathogenic microorganisms. In case of *Staphylococcus aureus* ATCC 29213 strain, we could link the level of AMPs in the tears to the antibacterial activity; reduced level of AMPs in the tear of patients with DM made this sample type the least effective. The reduced antimicrobial activity of tears caused by the altered level of AMPs may be the possible reason of the higher ocular infection rate in patients with DM.

## 4. Materials and Methods

### 4.1. Collection of Tear Samples

Tear collection was carried out using sterile glass capillary tubes (VWR Ltd., Radnor, PA, USA) without local anesthesia or stimulation [57]. Sample collection complied with the guidelines of the Helsinki Declaration and ethical approval was obtained from the University of Debrecen Ethics Committee (DEOEC RKEB/IKEB 3899-2013), while the subjects gave informed written consent. Non-stimulated tears samples were collected from each subject as described before [58]. In total, 35 donors were recruited into this study. In those cases where the sample collection was possible from both eyes, the collected tears were considered as different samples. Tears were collected from 13 eyes of 9 healthy individuals (5 male, 4 female subjects, mean age: 71 ± 9 years), from 10 eyes of 10 patients with diabetes without the sign of retinopathy (DM group, 4 male, 6 female subjects, mean age: 64 ± 10 years), from 6 eyes of 6 patients with non-proliferative retinopathy (NPDR group, 3 male, 3 female subject, mean age: 63 ± 14 years) and from 10 eyes of 10 patients with proliferative retinopathy (PDR group, 5 male, 5 female subject, mean age: 64 ± 8 years). There was no significant difference between the mean age in the studied groups.

### 4.2. Antibacterial Activity Analysis

Equal amounts of tears from each donor eyes were used for the antibacterial activity analysis. Pools were created from the samples of healthy controls and patients from DM, NPDR and PDR groups. For antibacterial studies, three pathogenic bacterial strains were selected: *Escherichia coli* ATCC 26922 strain, *Pseudomonas aeruginosa* ATCC 27853 strain and *Staphylococcus aureus* ATCC 29213 strain. The bacterial strains were cultured on Columbia blood agar (Oxoid Ltd., Basingstoke, UK) with 5% sheep blood. One colony from each bacterial strain were transferred to 5 mL sterile Luria–Bertani broth (LB) medium (Sigma-Aldrich, St. Louis, MO, USA) and incubated at 37 °C overnight. The bacteria suspensions were dissolved with fresh LB medium to the absorbance value of the pure LB at 620 nm wavelength. The samples were assembled on a 96-well microtiter plate in triplicates., and 100 µL pure LB with 10 µL physiological saline was used as blank. In addition, 100 µL diluted bacterial suspension with 10 µL physiological saline was used as growth control while the bacteriostatic effect of tears was analyzed by introducing 5 µL tear sample and 5µL physiological saline to 100 µL diluted bacterial suspension. Bacterial growth was monitored in every 30 min for 10 h by reading the absorbance of the samples at 620 nm wavelength with a Labsystems Multiskan MS plate reader (InterLabsystems Ltd., Budapest, Hungary). The microtiter plate was incubated at 37 °C during the whole experiment.

### 4.3. Sample Preparation for Mass Spectrometry

The protein concentration of tear samples was determined with the Bradford method [59]. Sample grouping (blocking) was carried out to avoid introduction of systematic bias and to maximize the ability to detect quantitative changes between groups [60]. Each group contained one randomly selected tear sample from healthy controls and one from patients with DM, NPDR or PDR, respectively. The four samples belonging to the newly created groups were processed as one batch on the same day and analyzed using the same conditions. 

The trypsin digestion of tear proteins was done as previously described [15]. Briefly, tear proteins were denatured with 6 M urea (Bio-Rad, Hercules, CA, USA) for 30 min; thereafter, reduced with 10 mM dithiothreitol (Bio-Rad, Hercules, CA, USA) at 56 °C for 60 min and further alkylated with 20 mM iodoacetamide (Bio-Rad, Hercules, CA, USA) in the dark for 45 min. Before trypsin digestion, samples were diluted with 25 mM ammonium bicarbonate (Sigma-Aldrich, St. Louis, MO, USA) to decrease the urea concentration to 1 M. Trypsin digestion was performed at 37 °C overnight by adding MS-grade modified trypsin (ABSciex, Framingham, MA, USA) in 1:25 enzyme to protein ratio. The digested tear proteins were dried in speed-vac and dissolved in 1% formic acid. The samples were desalted with C18 ZipTip (Merck Millipore, Darmstadt, Germany), dried and re-dissolved in 10 µL 1% formic acid before targeted mass spectrometry analyses. Quantification of peptides was undertaken by spiking well-defined amounts of their custom-synthesized, stable isotope-labeled (SIL) unpurified peptide analogues (JPT Peptide Technologies GmbH, Berlin, Germany) into the tryptic digest of each individual tear sample, immediately before the analyses. 

### 4.4. SRM-Based Targeted Mass Spectrometry Analysis

The peptide selection for the analyzed proteins was performed based on our previous study [15]. Briefly, the amino acid sequences of the selected proteins were collected from the UniProt database and in silico trypsin digestion was performed. Peptides with >95% cleavage probability were subjected to BLASTp analysis in order to identify the unique, protein specific peptides. The VTMLISGR, HVAYIIR and GLSTESILIPR peptides for lipocalin-1; the CGLVPVLAENYK and CLAENAGDVAFVK peptides for lactotransferrin; the QELNPLK and SILLTEQALAK peptides for extracellular glycoprotein lacritin; the GISLANWMCLAK and WESGYNTR peptides for lysozyme-C; the QIFGDYK peptide for lipophylin A; the SYSCQVTHEGSTVEK peptide for Ig λ-chain C region; the YTACLCDDNPK and TVQIAAVVDVIR peptides for prolactin inducible protein; the DYIEFNK; the IDVHWTR and DYIEFNK peptides for Zn α2 glycoprotein; the LADGGATNQGR and LASAYGAR peptides for galectin 3-binding protein and the ENAGEDPGLAR peptide for dermcidin were used. SRM transitions were designed for the selected peptides with the Skyline software [61], and all transitions were checked on the tear samples of healthy volunteers. The best two transitions per peptide were selected for the further analysis [15].

All samples were analyzed with a 4000 QTRAP (ABSciex, Framingham, MA, USA) mass spectrometer using a Nano Spray Micro Ion source controlled by the Analyst software (version 1.4.2; ABSciex, Framingham, MA, USA) in triplicates. The digested peptides were separated using an Easy nLC II nanoHPLC system (Bruker, Billerica, MA, USA). Then, 5 µg of total tear protein spiked with a fixed amount of unpurified SIL peptides were injected onto the Zorbax 300SB-C18 precolumn (5 × 0.3 mm, 5 µm particle size; Agilent Technologies Inc., Santa Clara, CA, USA) and further separated on a Zorbax 300SB-C18 analytical column (150 mm × 75 µm, 3.5 µm particle size; Agilent Technologies Inc., Santa Clara, CA, USA). Solvent A was 0.1% formic acid in LC water, solvent B was LC acetonitrile (Sigma-Aldrich, St. Louis, MO, USA) containing 0.1% formic acid. The flow rate was set to 300 nL/min during the separation. A 30 min acetonitrile/water gradient was used with an increase in solvent B concentration from 0 to 100% in 15 min. The eluate from the LC column was ionized using electrospray ionization with 2800 V spray voltage and positive ion mode SRM spectra were recorded. Other acquisition parameters were as follows: the ion source gas was 50 psi; the curtain gas was 20 psi, and the source temperature was 70 °C, and the cycle time was 2.5 s.

### 4.5. Data and Statistical Analysis

SRM data were evaluated using the Skyline software [61]. The acquired data were uploaded to the Panorama website (https://panoramaweb.org/University%20of%20Debrecen/Diab_tear_SRM/project-begin.view? accessed on 13 March 2021) and are publicly available. The area under the curve (AUC) values of the SRM spectra were calculated with the Skyline software. The primary AUC ratios were transformed to MSstats (version 2.0) by an in-house developed software [62]. After the normalization, based on the SIL standard peptides and log2 transformation of data, group differences were examined by a mixed-effect analysis of variance, modelling group specific variance as fixed effects and subject level deviations as random effects. After the model estimation, we used post hoc Tukey tests to characterize the group-pair differences by Student’s-t statistic and the corresponding *p*-values. 

Data from the antibacterial analysis experiments were collected into a Microsoft Excel worksheet, and statistical analysis was carried out with SPSS Statistics 25 software (IBM). Data were subjected to Shapiro–Wilk normality test and as far as they have shown non-normal distribution, the non-parametric Mann–Whitney U-test was used to examine the differences between the studied groups.

Statistical significance was set to *p* < 0.05 in all cases.

## 5. Conclusions

In this pilot study, we have shown the altered level of some AMPs in the tears of healthy controls and patients with diabetes along with the antimicrobial activity of tear samples on three examined pathogenic bacterial strains. A disease stage-specific inhibitory effect was observed in case of *Staphylococcus aureus* ATCC 29213 strain; tears from patients with DM have shown the lowest antimicrobial activity while tears from patients with PDR had the highest antimicrobial activity among the studied groups. No antimicrobial activity was observed in case of *Escherichia coli* ATCC 26922 strain, and a non-disease specific inhibitory effect of tears was observed in case of *Pseudomonas aeruginosa* ATCC 27853 strain. The changes in the level of the major tear AMPs revealed by SRM-based targeted mass spectrometry analyses may be responsible for the observed disease stage-specific antimicrobial activity of tears. The possible reason for the altered antimicrobial activity against the studied bacterial strains may be the different composition and structure of the cell wall of the Gram-positive and -negative species.

Our data suggest that the altered composition of tears may be the reason behind the altered antimicrobial activity, and hence, a possible cause of the higher eye infection rate observed in patients with diabetes. Further studies are needed to confirm the link between the altered tear proteome and the higher infection rate. Identifying the proteins required for the antimicrobial activity can help in designing new therapeutic intervention possibilities to prevent the ocular infection in patients with diabetes. 

## Figures and Tables

**Table 1:** Antibacterial activity of tears against *Staphylococcus aureus* ATCC 29213 strain. The calculated *p*-values from the Mann–Whitney U-test are indicated. Bold values represent significant differences between the groups (*p* < 0.05).

Time (h)	Control vs. Healthy	Control vs. DM	Control vs. NPDR	Control vs. PDR	Healthy vs. DM	Healthy vs. NPDR	Healthy vs. PDR	DM vs. NPDR	DM vs. PDR	NPDR vs. PDR
0	0.099	0.268	0.099	0.099	0.369	1.000	1.000	0.369	0.369	1.000
0.5	**0.049**	**0.049**	0.077	**0.046**	0.261	0.261	0.346	0.822	0.637	0.637
1	**0.046**	**0.046**	**0.046**	**0.049**	0.099	0.099	0.105	0.361	0.637	0.637
1.5	**0.043**	**0.043**	**0.043**	**0.043**	0.796	0.099	**0.043**	0.361	0.068	0.068
2	0.268	0.653	0.105	**0.049**	0.197	0.500	0.121	**0.043**	**0.046**	**0.046**
2.5	**0.049**	**0.049**	**0.049**	**0.049**	**0.049**	0.184	**0.049**	0.184	**0.049**	**0.049**
3	**0.049**	**0.049**	**0.049**	**0.046**	**0.049**	0.658	**0.046**	0.127	**0.046**	**0.046**
3.5	**0.046**	**0.046**	**0.046**	**0.046**	**0.049**	0.658	**0.049**	0.077	**0.049**	**0.049**
4	**0.046**	**0.046**	**0.046**	**0.046**	**0.049**	**0.049**	**0.049**	0.077	**0.049**	**0.049**
4.5	**0.049**	0.275	0.127	**0.049**	**0.049**	**0.049**	**0.049**	0.275	**0.049**	**0.049**
5	**0.049**	0.275	0.827	**0.049**	**0.049**	**0.049**	0.127	0.275	**0.049**	**0.049**
5.5	**0.049**	0.275	0.275	**0.049**	**0.049**	**0.049**	0.827	0.376	**0.049**	**0.049**
6	**0.049**	0.827	0.275	0.275	**0.049**	**0.049**	0.513	0.275	0.275	0.275
6.5	0.827	0.827	0.127	0.513	0.827	0.513	0.513	0.513	0.513	0.513
7	**0.049**	**0.049**	**0.049**	**0.049**	**0.049**	**0.049**	**0.049**	0.513	**0.049**	**0.049**
7.5	**0.049**	0.275	**0.049**	0.275	**0.049**	**0.049**	**0.049**	0.513	**0.049**	**0.049**
8	**0.049**	0.275	0.275	0.127	**0.049**	**0.049**	0.127	0.513	0.127	0.127
8.5	**0.049**	0.268	0.376	0.127	**0.049**	**0.049**	**0.049**	0.507	**0.046**	**0.049**
9	**0.049**	0.127	0.127	**0.049**	**0.049**	**0.049**	**0.049**	0.827	**0.049**	**0.049**
9.5	**0.049**	0.275	0.275	**0.049**	**0.049**	**0.049**	0.275	0.513	**0.049**	**0.049**
10	0.268	0.268	0.268	0.268	0.127	**0.049**	0.275	0.658	0.275	0.275

**Table 2:** Antibacterial activity of tears against *Escherichia coli* ATCC 26922. The calculated *p*-values from the Mann–Whitney U-test are indicated. Bold values represent significant differences between the groups (*p* < 0.05).

Time (h)	Control vs. Healthy	Control vs. DM	Control vs. NPDR	Control vs. PDR	Healthy vs. DM	Healthy vs. NPDR	Healthy vs. PDR	DM vs. NPDR	DM vs. PDR	NPDR vs. PDR
0	**0.046**	0.197	0.268	0.369	0.369	0.513	0.513	0.507	0.825	0.275
0.5	**0.049**	**0.046**	0.077	0.127	0.105	0.513	0.658	0.507	0.507	0.827
1	0.077	0.127	0.072	0.268	0.275	0.369	0.121	0.268	0.637	0.116
1.5	**0.046**	**0.046**	**0.046**	**0.046**	0.513	0.827	0.513	0.658	0.376	0.184
2	**0.049**	**0.049**	**0.049**	**0.049**	0.376	0.658	0.513	0.827	0.127	0.077
2.5	**0.049**	**0.049**	**0.049**	**0.049**	0.127	0.275	0.513	0.827	0.513	0.658
3	0.275	0.127	0.513	0.268	**0.049**	0.275	0.268	**0.049**	0.507	0.268
3.5	0.275	0.827	0.127	0.275	0.275	0.513	0.275	0.127	0.513	0.827
4	0.127	**0.049**	**0.049**	**0.049**	0.184	0.658	0.275	0.184	0.827	0.275
4.5	0.513	0.275	0.827	0.275	**0.049**	0.827	0.275	0.184	0.827	0.275
5	0.658	**0.049**	0.127	**0.049**	0.127	0.658	**0.049**	0.275	0.827	**0.049**
5.5	0.827	0.127	0.827	0.275	0.275	0.827	0.376	0.184	0.827	0.275
6	0.275	0.127	1.000	0.127	0.127	0.275	**0.049**	0.127	0.827	0.127
6.5	0.513	0.275	0.827	0.513	0.127	0.513	0.261	0.275	0.827	0.513
7	0.127	0.827	0.513	0.268	**0.049**	0.127	**0.046**	**0.049**	**0.046**	0.046
7.5	0.127	0.658	0.275	0.275	0.127	0.275	**0.049**	0.275	0.275	0.275
8	0.127	0.827	0.513	0.827	0.275	0.275	0.275	0.275	0.275	0.275
8.5	0.513	0.827	0.827	0.127	0.127	0.275	**0.049**	0.275	0.275	**0.049**
9	**0.046**	**0.049**	**0.049**	**0.049**	**0.046**	**0.046**	**0.046**	**0.049**	**0.049**	**0.049**
9.5	0.827	0.275	0.513	0.275	0.513	0.513	0.127	0.658	0.513	0.275
10	0.827	**0.049**	0.127	**0.049**	**0.049**	**0.049**	**0.049**	0.184	0.184	0.077

**Table 3:** Antibacterial activity of tears against *Pseudomonas aeruginosa* ATCC 27853 strain. The calculated *p*-values from the Mann–Whitney U-test are indicated. Bold values represent significant differences between the groups (*p* < 0.05).

Time (h)	Control vs. Healthy	Control vs. DM	Control vs. NPDR	Control vs. PDR	Healthy vs. DM	Healthy vs. NPDR	Healthy vs. PDR	DM vs. NPDR	DM vs. PDR	NPDR vs. PDR
0	**0.046**	0.127	0.127	**0.046**	0.487	0.268	0.500	1.000	0.507	0.507
0.5	**0.046**	**0.046**	**0.046**	**0.046**	1.000	0.513	0.513	0.827	0.261	0.376
1	**0.046**	**0.046**	**0.046**	**0.034**	0.658	0.658	0.121	0.261	**0.037**	**0.037**
1.5	**0.049**	**0.046**	**0.049**	**0.046**	0.817	0.822	0.369	0.487	0.099	**0.046**
2	**0.049**	**0.049**	**0.046**	**0.049**	0.658	0.637	0.513	0.105	0.261	**0.046**
2.5	0.077	0.184	0.077	**0.049**	0.500	0.500	**0.049**	0.658	**0.049**	**0.049**
3	0.376	0.658	0.658	**0.049**	0.827	1.000	**0.049**	0.658	**0.049**	**0.049**
3.5	1.000	0.513	0.827	**0.049**	1.000	0.658	**0.049**	0.275	**0.049**	**0.049**
4	0.822	0.513	0.658	**0.049**	0.827	0.827	**0.049**	0.275	**0.049**	0.077
4.5	0.105	0.127	0.513	**0.049**	0.825	0.825	**0.046**	0.275	**0.049**	**0.049**
5	0.827	0.275	0.275	**0.046**	0.275	0.513	**0.046**	0.127	**0.046**	0.121
5.5	0.513	0.827	0.275	**0.049**	0.827	0.513	0.275	0.513	**0.049**	0.275
6	**0.049**	**0.049**	**0.049**	**0.049**	0.275	0.275	0.077	0.658	0.658	0.658
6.5	0.513	**0.049**	0.827	**0.049**	0.513	0.513	**0.049**	0.513	**0.049**	0.275
7	**0.049**	**0.049**	**0.049**	**0.049**	0.275	0.827	**0.049**	0.275	**0.049**	**0.049**
7.5	**0.049**	**0.049**	**0.049**	**0.049**	0.513	**0.049**	0.127	**0.049**	0.077	0.827
8	0.513	**0.049**	**0.049**	0.513	0.827	0.127	0.513	0.127	0.827	0.275
8.5	**0.049**	**0.049**	**0.049**	**0.049**	0.513	0.827	0.827	0.513	0.275	0.513
9	**0.049**	**0.049**	**0.049**	**0.049**	0.827	0.513	0.827	0.827	0.827	0.513
9.5	**0.049**	**0.049**	**0.049**	**0.049**	0.513	0.513	0.827	0.513	0.513	0.827
10	**0.049**	**0.049**	**0.049**	**0.049**	0.827	0.513	0.513	0.827	0.827	0.658

## Data Availability

The acquired SRM data are publicly available at the Panorama website (https://panoramaweb.org/University%20of%20Debrecen/Diab_tear_SRM/project-begin.view? accessed on 13 March 2021).

## Supplementary Materials

The following are available online at https://www.mdpi.com/article/10.3390/pathogens10070883/s1. Table S1: Antimicrobial activity of tears against *Staphylococcus aureus* ATCC 29213 strain. Table S2: Antimicrobial activity of tears against *Escherichia coli* ATCC 26922 strain. Table S3: Antimicrobial activity of tears against *Pseudomonas aeruginosa* ATCC 27853 strain.
