# Supplementary material for: Reduced Level of Tear Antimicrobial and Immunomodulatory Proteins as a Possible Reason for Higher Ocular Infections in Diabetic Patients
Source: Pathogens. 2021 Jul 12;10(7):883. doi: 10.3390/pathogens10070883 (PMC8308669; doi:10.3390/pathogens10070883)
Supplement: Supplementary file 1 [file pathogens-10-00883-s001.zip › Table S2.pdf]

Time (h)	Control		Healthy		DM		NPDR		PDR		P – value (Mann Whitney U-test)									
	Mean absorbance	SD	Mean absorbance	SD	Mean absorbance	SD	Mean absorbance	SD	Mean absorbance	SD	Control - Healthy	Control – DM	Control - NPDR	Control – PDR	Healthy – DM	Healthy – NPDR	Healthy – PDR	DM – NPDR	DM – PDR	NPDR – PDR
0	0,014	0,002	0,019	0,003	0,018	0,004	0,022	0,008	0,017	0,006	**0,046**	0,197	0,268	0,369	0,369	0,513	0,513	0,507	0,825	0,275
0,5	0,019	0,002	0,026	0,001	0,024	0,001	0,026	0,005	0,028	0,009	**0,049**	**0,046**	0,077	0,127	0,105	0,513	0,658	0,507	0,507	0,827
1	0,038	0,004	0,045	0,003	0,043	0,002	0,046	0,003	0,042	0,002	0,077	0,127	0,072	0,268	0,275	0,369	0,121	0,268	0,637	0,116
1,5	0,073	0,003	0,085	0,005	0,084	0,003	0,086	0,005	0,082	0,002	**0,046**	**0,046**	**0,046**	**0,046**	0,513	0,827	0,513	0,658	0,376	0,184
2	0,123	0,005	0,139	0,005	0,142	0,004	0,142	0,005	0,136	0,002	**0,049**	**0,049**	**0,049**	**0,049**	0,376	0,658	0,513	0,827	0,127	0,077
2,5	0,191	0,004	0,202	0,008	0,220	0,019	0,217	0,017	0,213	0,017	**0,049**	**0,049**	**0,049**	**0,049**	0,127	0,275	0,513	0,827	0,513	0,658
3	0,254	0,023	0,269	0,022	0,227	0,005	0,249	0,010	0,231	0,028	0,275	0,127	0,513	0,268	**0,049**	0,275	0,268	**0,049**	0,507	0,268
3,5	0,262	0,030	0,312	0,040	0,274	0,012	0,304	0,022	0,285	0,048	0,275	0,827	0,127	0,275	0,275	0,513	0,275	0,127	0,513	0,827
4	0,352	0,016	0,311	0,031	0,271	0,024	0,302	0,017	0,275	0,033	0,127	**0,049**	**0,049**	**0,049**	0,184	0,658	0,275	0,184	0,827	0,275
4,5	0,310	0,049	0,305	0,024	0,271	0,018	0,300	0,031	0,277	0,042	0,513	0,275	0,827	0,275	**0,049**	0,827	0,275	0,184	0,827	0,275
5	0,341	0,003	0,334	0,014	0,303	0,024	0,326	0,012	0,301	0,006	0,658	**0,049**	0,127	**0,049**	0,127	0,658	**0,049**	0,275	0,827	**0,049**
5,5	0,343	0,026	0,332	0,037	0,301	0,023	0,328	0,028	0,301	0,045	0,827	0,127	0,827	0,275	0,275	0,827	0,376	0,184	0,827	0,275
6	0,323	0,017	0,330	0,012	0,299	0,023	0,322	0,021	0,293	0,014	0,275	0,127	1,000	0,127	0,127	0,275	**0,049**	0,127	0,827	0,127
6,5	0,339	0,014	0,359	0,038	0,322	0,023	0,342	0,028	0,318	0,041	0,513	0,275	0,827	0,513	0,127	0,513	0,261	0,275	0,827	0,513
7	0,305	0,020	0,329	0,010	0,301	0,003	0,319	0,004	0,291	0,013	0,127	0,827	0,513	0,268	**0,049**	0,127	**0,046**	**0,049**	**0,046**	0,046
7,5	0,330	0,022	0,371	0,022	0,336	0,026	0,354	0,022	0,323	0,017	0,127	0,658	0,275	0,275	0,127	0,275	**0,049**	0,275	0,275	0,275
8	0,367	0,042	0,415	0,042	0,375	0,038	0,391	0,029	0,361	0,033	0,127	0,827	0,513	0,827	0,275	0,275	0,275	0,275	0,275	0,275
8,5	0,361	0,049	0,369	0,019	0,336	0,021	0,349	0,013	0,320	0,012	0,513	0,827	0,827	0,127	0,127	0,275	**0,049**	0,275	0,275	**0,049**
9	0,396	0,027	0,357	0,006	0,324	0,002	0,338	0,009	0,307	0,012	**0,046**	**0,049**	**0,049**	**0,049**	**0,046**	**0,046**	**0,046**	**0,049**	**0,049**	**0,049**
9,5	0,389	0,016	0,401	0,051	0,358	0,041	0,369	0,038	0,341	0,043	0,827	0,275	0,513	0,275	0,513	0,513	0,127	0,658	0,513	0,275
10	0,417	0,024	0,419	0,007	0,373	0,004	0,389	0,020	0,356	0,017	0,827	**0,049**	0,127	**0,049**	**0,049**	**0,049**	**0,049**	0,184	0,184	0,077

Table S2: Antimicrobial activity of tears against *Escherichia coli* ATCC 26922 strain. The mean absorbance and SD values are indicated along with the calculated p-values from the Mann Whitney U-test. Bold values represent significant differences between the groups ($p \leq 0,05$).
