# Supplementary material for: Reduced Level of Tear Antimicrobial and Immunomodulatory Proteins as a Possible Reason for Higher Ocular Infections in Diabetic Patients
Source: Pathogens. 2021 Jul 12;10(7):883. doi: 10.3390/pathogens10070883 (PMC8308669; doi:10.3390/pathogens10070883)
Supplement: Supplementary file 1 [file pathogens-10-00883-s001.zip › Table S3.pdf]

Time (h)	Control		Healthy		DM		NPDR		PDR		P – value (Mann Whitney U-test)									
	Mean absorbance	SD	Mean absorbance	SD	Mean absorbance	SD	Mean absorbance	SD	Mean absorbance	SD	Control - Healthy	Control – DM	Control - NPDR	Control – PDR	Healthy – DM	Healthy – NPDR	Healthy – PDR	DM – NPDR	DM – PDR	NPDR – PDR
0	0,004	0,002	0,011	0,005	0,009	0,004	0,009	0,005	0,011	0,002	**0,046**	0,127	0,127	**0,046**	0,487	0,268	0,500	1,000	0,507	0,507
0,5	0,006	0,001	0,013	0,004	0,012	0,002	0,011	0,003	0,014	0,001	**0,046**	**0,046**	**0,046**	**0,046**	1,000	0,513	0,513	0,827	0,261	0,376
1	0,008	0,001	0,015	0,002	0,015	0,001	0,014	0,002	0,017	0,000	**0,046**	**0,046**	**0,046**	**0,034**	0,658	0,658	0,121	0,261	**0,037**	**0,037**
1,5	0,014	0,002	0,021	0,003	0,021	0,001	0,020	0,001	0,023	0,001	**0,049**	**0,046**	**0,049**	**0,046**	0,817	0,822	0,369	0,487	0,099	**0,046**
2	0,023	0,002	0,031	0,003	0,031	0,001	0,030	0,001	0,032	0,001	**0,049**	**0,049**	**0,046**	**0,049**	0,658	0,637	0,513	0,105	0,261	**0,046**
2,5	0,042	0,002	0,046	0,002	0,045	0,003	0,045	0,002	0,052	0,002	0,077	0,184	0,077	**0,049**	0,500	0,500	**0,049**	0,658	**0,049**	**0,049**
3	0,064	0,003	0,066	0,003	0,065	0,004	0,066	0,005	0,077	0,004	0,376	0,658	0,658	**0,049**	0,827	1,000	**0,049**	0,658	**0,049**	**0,049**
3,5	0,093	0,003	0,093	0,004	0,091	0,005	0,096	0,007	0,110	0,009	1,000	0,513	0,827	**0,049**	1,000	0,658	**0,049**	0,275	**0,049**	**0,049**
4	0,137	0,003	0,135	0,007	0,133	0,010	0,138	0,011	0,159	0,014	0,822	0,513	0,658	**0,049**	0,827	0,827	**0,049**	0,275	**0,049**	0,077
4,5	0,215	0,007	0,203	0,010	0,200	0,012	0,208	0,014	0,243	0,019	0,105	0,127	0,513	**0,049**	0,825	0,825	**0,046**	0,275	**0,049**	**0,049**
5	0,314	0,011	0,309	0,014	0,296	0,016	0,323	0,017	0,375	0,033	0,827	0,275	0,275	**0,046**	0,275	0,513	**0,046**	0,127	**0,046**	0,121
5,5	0,396	0,010	0,409	0,026	0,390	0,037	0,419	0,030	0,454	0,035	0,513	0,827	0,275	**0,049**	0,827	0,513	0,275	0,513	**0,049**	0,275
6	0,407	0,012	0,431	0,008	0,442	0,010	0,440	0,010	0,445	0,011	**0,049**	**0,049**	**0,049**	**0,049**	0,275	0,275	0,077	0,658	0,658	0,658
6,5	0,418	0,014	0,434	0,019	0,447	0,006	0,433	0,039	0,503	0,053	0,513	**0,049**	0,827	**0,049**	0,513	0,513	**0,049**	0,513	**0,049**	0,275
7	0,427	0,016	0,505	0,014	0,486	0,021	0,505	0,010	0,580	0,030	**0,049**	**0,049**	**0,049**	**0,049**	0,275	0,827	**0,049**	0,275	**0,049**	**0,049**
7,5	0,513	0,027	0,594	0,023	0,608	0,008	0,652	0,021	0,647	0,029	**0,049**	**0,049**	**0,049**	**0,049**	0,513	**0,049**	0,127	**0,049**	0,077	0,827
8	0,571	0,014	0,596	0,069	0,635	0,023	0,672	0,030	0,601	0,103	0,513	**0,049**	**0,049**	0,513	0,827	0,127	0,513	0,127	0,827	0,275
8,5	0,586	0,017	0,446	0,033	0,494	0,082	0,469	0,024	0,438	0,052	**0,049**	**0,049**	**0,049**	**0,049**	0,513	0,827	0,827	0,513	0,275	0,513
9	0,627	0,011	0,384	0,044	0,397	0,055	0,392	0,007	0,391	0,041	**0,049**	**0,049**	**0,049**	**0,049**	0,827	0,513	0,827	0,827	0,827	0,513
9,5	0,642	0,032	0,382	0,073	0,385	0,021	0,402	0,037	0,399	0,052	**0,049**	**0,049**	**0,049**	**0,049**	0,513	0,513	0,827	0,513	0,513	0,827
10	0,655	0,051	0,367	0,053	0,384	0,045	0,392	0,021	0,400	0,022	**0,049**	**0,049**	**0,049**	**0,049**	0,827	0,513	0,513	0,827	0,827	0,658

Table 3: Antimicrobial activity of tears against *Pseudomonas aeruginosa* ATCC 27853 strain. The mean absorbance and SD values are indicated along with the calculated p-values from the Mann Whitney U-test. Bold values represent significant differences between the groups (p≤0,05).
